# Hand disinfection in a neonatal intensive care unit: continuous electronic monitoring over a one-year period

**DOI:** 10.1186/1471-2334-12-248

**Published:** 2012-10-08

**Authors:** Onno K Helder, Johannes B van Goudoever, Wim C J Hop, Johannes Brug, René F Kornelisse

**Affiliations:** 1 Department of Pediatrics, Division of Neonatology, Erasmus MC - Sophia Children’s Hospital, Erasmus University Medical Center, PO Box 2060, room SK-1286, Rotterdam, 3000 CB, The Netherlands; 2 Department of Pediatrics, VU University Medical Center Amsterdam, Amsterdam, The Netherlands; 3 Department of Pediatrics, Emma Children’s Hospital, AMC Amsterdam, Amsterdam, The Netherlands; 4 Department of Biostatistics, Erasmus University Medical Center Rotterdam, Rotterdam, The Netherlands; 5 EMGO Institute for Health and Care Research, VU University Medical Center Amsterdam, Amsterdam, The Netherlands

## Abstract

**Background:**

Good hand hygiene compliance is essential to prevent nosocomial infections in healthcare settings. Direct observation of hand hygiene compliance is the gold standard but is time consuming. An electronic dispenser with built-in wireless recording equipment allows continuous monitoring of its usage. The purpose of this study was to monitor the use of alcohol-based hand rub dispensers with a built-in electronic counter in a neonatal intensive care unit (NICU) setting and to determine compliance with hand hygiene protocols by direct observation.

**Methods:**

A one-year observational study was conducted at a 27 bed level III NICU at a university hospital. All healthcare workers employed at the NICU participated in the study. The use of bedside dispensers was continuously monitored and compliance with hand hygiene was determined by random direct observations.

**Results:**

A total of 258,436 hand disinfection events were recorded; i.e. a median (interquartile range) of 697 (559–840) per day. The median (interquartile range) number of hand disinfection events performed per healthcare worker during the day, evening, and night shifts was 13.5 (10.8 - 16.7), 19.8 (16.3 - 24.1), and 16.6 (14.2 - 19.3), respectively. In 65.8% of the 1,168 observations of patient contacts requiring hand hygiene, healthcare workers fully complied with the protocol.

**Conclusions:**

We conclude that the electronic devices provide useful information on frequency, time, and location of its use, and also reveal trends in hand disinfection events over time. Direct observations offer essential data on compliance with the hand hygiene protocol. In future research, data generated by the electronic devices can be supplementary used to evaluate the effectiveness of hand hygiene promotion campaigns.

## Background

Staff compliance with hand hygiene protocols in neonatal intensive care units (NICUs) is highly important to limit the spread of pathogens by the hands of healthcare workers and thus to prevent nosocomial infections [1]. Incidences of bloodstream infections in infants admitted to NICUs currently range from 12% to 53% [2]. There is evidence that improved hand hygiene in NICU settings results in infection reduction [3]. Hand hygiene performance used to be determined by direct observation, but electronic counting methods have been introduced as an alternative.

Three previous studies used bedside electronic counting devices designed to record hand rub dispenser lever-presses [4-6]. Cheng et al. and Marra et al. concluded that unobtrusive measurement by electronic devices results in more objective data since direct observations might influence hand hygiene compliance behaviour [4,6]. Boyce et al. found that hand disinfection was more frequent performed in the adult intensive care setting than in the general medical ward setting [5]. However, these studies had some limitations: data were collected over a relatively short period and detailed information on hand hygiene events distribution over the day was not provided.

We present the results of a study whose objectives were: [1] to monitor the use of alcohol-based hand rub dispensers with a built-in electronic counter in our NICU over a one-year period; [2] to determine compliance with hand hygiene by direct observations; and [3] to compare numbers of hand disinfection events during different shifts and determine differences in distribution of these events over the day.

## Methods

### Setting

This prospective observational study was performed from January 1^st^ to December 31^st^ of 2008 in a 27-bed level III NICU at a university hospital in the Netherlands. The NICU is organized into three identical sub-units with nine beds each.

Appropriate hand hygiene is considered an important safety issue which is dealt with in education programs since June 2005 [2]. The institutional hand hygiene protocol used during the study period dictated that hand hygiene had to be applied before patient and after patient contact as well as before and after invasive procedures. The currently used ‘My five moments for hand hygiene’ approach had not yet been published at the time [7]. Hand alcohol is generally preferred to soap. The only exceptions are visible soiling of the hands, bathroom visits, and the presence of pathogens that are immune for hand alcohol, such as Clostridium and some gastroenteritis viruses. At least 3 ml of hand alcohol should be applied to rub hands for at least 30 seconds. Hand alcohol dispensers (Baktosept E, Bode Chemie GmbH, Hamburg, Germany) are available at each bedside. Furthermore, non-sterile gloves must be worn when there is a risk of exposure to a patient’s body fluid. Then, hand disinfection is applied before and after glove use. In addition, two sinks with soap dispensers are located next to the nurses’ station. One of these sinks also has a hand alcohol dispenser (Sterillium, Bode Chemie GmbH, Hamburg, Germany), which is exclusively used for surgical hand disinfection. However, Sterillium is approved for both hygienic and surgical hand disinfection. This dispenser is not provided with an electronic counting device.

### Data collection

All 27 wall-mounted alcohol-based hand rub bedside dispensers have a concealed electronic counter and wireless transmitting equipment (ComSens NewCompliance, Delft, the Netherlands). The counter documents date and time of each individual use of the dispenser. The system does not allow distinguishing between categories of healthcare workers; data are collected anonymously. Each lever-press generates a click of the sensor; a click within a 2-second period of the previous click was considered as one hand disinfection event [5,6]. All dispensers delivered 1.8 ml per full lever-press. Data collected from the dispensers were transmitted to a computer-linked receiver. The study population for which dispenser use was recorded consisted of healthcare workers only (nurses, nurse practitioners, nursing assistants, and physicians). Parents and visitors were strongly encouraged to wash their hands with soap only.

The frequency of hand disinfection events was expressed in two ways: the daily median [interquartile range (IQR)] number of hand disinfection events per bedside; and the daily median (IQR) number of hand disinfection events per healthcare worker. The day shift, evening shift and night shift extended from 8:00 h to 16:00 h; from 16:00 h to 23:00 h; and from 23:00 h to 8:00 h of the next day, respectively.

Additionally, we randomly observed healthcare workers’ compliance with the hand hygiene protocol, using a tool described in a previous study [2]. Failure to disinfect hands before or after patient contact, and before or after invasive tasks was recorded as non-compliance. Data were collected during thirty 60-minute observation sessions in each sub-unit, from 8:00 h to 22:00 h on weekdays. Hygienic performance starts at each new patient contact, so in theory a healthcare worker can perform more than one care sequence during an observation period. Observations were carried out from January to February 2008 and from May to June 2008, simultaneously with hand dispenser recordings. Immediate life-saving interventions were excluded from analysis [2]. Three trained researchers and the prevention expert (OH) independently observed hand hygiene events. Interobserver reliability assessed by Cohen’s Kappa was high (κ > 0.70).

The number of hand hygiene events for an ideal 100% compliance with hand hygiene was calculated (total sum of recorded hand disinfection events x 100/ compliance).

### Statistical analysis

Data are expressed as the median (IQR). The sign test served to compare numbers of hand disinfection events among shifts for each day. SPSS version 17.0 (SPSS, Chicago, IL) was used for analysis, and p < 0.05 (two-sided) was considered as significant.

The Institutional Review Board of the University Medical Center Rotterdam approved this study at August 23 2007.

## Results

During the one-year study period, a total of 717,445 lever-presses for all dispensers were recorded, equivalent to 258,436 hand disinfection events. The calculated median (IQR) number of hand disinfection events per day was 697 (559–840). The proportion of hand disinfection events during day shifts was 41.0%, which is significantly higher than that during evening shifts (34.9%) and night shifts (24.1%).

The median (IQR) daily number of healthcare workers who provided patient care was 44 (42–45), i.e. 34 nurses and 10 physicians and nurse practitioners. The distribution of both disciplines (median) during day, evening and night shifts was 14 vs. 7; 10 vs. 2; and 9 vs. 1, respectively. The average number of lever-presses per hand disinfection events was 2.8, which equals 5 ml hand alcohol if all lever-presses were fully completed.

**Figure 1:**
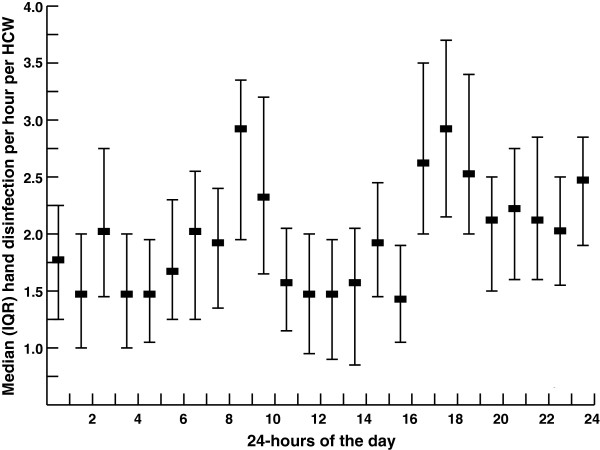
**Median (IQR) number of hand disinfection events per healthcare worker plotted for each hour of the day, calculated over the one-year study period.** Analysis of hand disinfection events per healthcare worker by hour of the day revealed a significant increase in hand disinfection events from 8:00 h to 10:00 h, which coincides with the start of the dayshift and medical assessments. Another increase was found from 16:00 h to 19:00 h, which correspondents with elevated activities before dinnertime (p < 0.001 for both). The number of hand disinfection events was relatively low from 10:00 h to 16:00 h.

The median (IQR) number of hand disinfection events per healthcare worker per day was 15.9 (13.1-19.3). In Figure 1 the numbers of hand disinfection events per healthcare worker are plotted for each hour of the day, calculated over the one-year study period.

The distributions for day shift, evening shift, and night shift are presented in Table 1. Differences between shifts were all statistically significant (p < 0.001). The median (IQR) number of hand disinfection events per patient-day was 27.6 (23.0-36.3).

**Table 1:** **Distribution of hand disinfection events per healthcare worker over the different shifts**

**Shift**	**Median (IQR**^**#**^**) hand disinfection eventsper healthcare worker**
Day shift	13.9 (10.8-16.7)
Evening shift	19.8 (16.3-24.1)
Night shift	16.6 (14.2-19.3)
Total day	15.9 (13.1-19.3)

^#^ IQR: interquartile range.

In total 1,168 direct observations of events requiring hand hygiene were analysed; in 65.8% of cases healthcare workers fully complied with the protocol. The interquartile range of compliance with hand hygiene determined at the separate observation days varied from 50% to 71.5%.

Adjusted for the 65.8% compliance rate, the counted number of hand disinfection events should increase by about 50% to approximately 375,000 hand hygiene disinfection events.

## Discussion

Electronic dispensers provided data trends on the frequency of hand disinfection events in a clinical setting over an extended period of time. The median number of 15.9 hand disinfection per healthcare worker per day in our study falls within the median 5.0-30.0 range reported by Boyce et al. [1].

Three studies measuring hand disinfection events by electronic dispensers expressed the outcome as hand disinfections per patient-day [5,6,8]. For a pediatric intensive care unit, a surgical intensive care unit and a general medical ward, the mean number was 41.2, 48.7 and 12.2, respectively [6]. Marra et al. reported a mean of 53.8 hand disinfections per patient-day in an adult medical-surgical intensive care unit; [6]. Another study performed in a general pediatric ward measured the amount of used hand alcohol and translated this into 47 hand rubs per patient-day [9]. McGluckin et al. reported a mean of 6.7 hand washings per patient-day in an inpatient rehabilitation unit [10]. We documented a median of 27.6 hand disinfection events per patient-day at our NICU. This relatively low number as compared to two of the studies mentioned above likely reflects our policy to provide care on indication. This approach takes into account the infants’ sleep-wake rhythm so that they can sleep longer, which improves recovery from previous interventions. This approach leads to fewer patient contacts.

Combining the electronically collected data and the observational data allows generating an additional tool to monitor hand hygiene practices. The calculated number of required hand disinfection events per day could be an incentive for healthcare workers to strive for and reach 100% compliance. However, this calculated number is ward-specific and may be only adhered to if conditions such as case mix, number of patient days, and patient-healthcare worker ratio, are comparable to conditions of the initial study period.

Additionally, we showed that hand hygiene performance followed a daily pattern: it was most intense after shift handover, and after dinnertime. The median number of hand disinfection events per healthcare worker during day shifts was lower than that during evening shifts. This is probably caused by the fact that the work floor during day shifts counts twice as many healthcare workers than during evening shifts; the number of patient contacts is likely not doubled. The slightly lower number of hand disinfection events per healthcare worker during night shifts in comparison to evening shifts might be explained by the fact that night shifts in general correlate negatively with hand hygiene compliance [11]. Additionally, in the night shifts there are fewer hand disinfection opportunities as healthcare workers only perform routine care and unavoidable interventions.

Direct observation of hygienic behavior is a well-known method to document hand hygiene compliance in a clinical setting. Nevertheless, it is time consuming, and knowing that they are observed may influence the healthcare workers’ behavior [4-6]. In contrast, the described electronic device unobtrusively records all hand disinfection events over an extended period of time. Furthermore, senior staff can motivate members of the healthcare team to improve their hand hygiene practices by relating the recorded number of hand hygiene events to the calculated number required for 100% compliance. Nevertheless, this device is not able to record non-compliance and the quality of hand disinfection. Non-compliance can be defined as failure to disinfect hands, lack of completeness of hand rubbing, or insufficient drying time. Applying both methods together therefore provides a more complete representation of hand hygiene practices.

This study had several limitations. The used type of dispenser is unable to detect whether dispenser use correlates with a defined hand disinfection opportunity. Second, this study was designed and performed before the ‘My five moments for hand hygiene’ approach was published [7]. Three of the five hand hygiene indications were measured: before patient contact, before invasive procedures, and after patient contact. The ‘My five moments for hand hygiene’ approach is nowadays considered the “gold standard” method to monitor hand hygiene compliance. We missed the 3^rd^ and 5^th^ moments: ‘after touching patient surroundings’ and after body fluid exposure risk. However, our hand hygiene protocol dictates that healthcare workers must wear gloves when at risk of exposure to a patient’s body fluid. They are also required to disinfect hands before and after glove use. Third, the variance of hand disinfection practices by individual healthcare workers was not documented. Furthermore, we also cannot rule out the possibility that parents or family occasionally used alcohol dispensers, although all NICU professionals instructed parents to wash their hands with soap only. NICU professionals did not report the use of hand alcohol by parents. In addition, healthcare workers also might have used hand alcohol at moments that are not corresponding to any indication for hand hygiene. This possible unnoticed use could have resulted in overestimation of hand hygiene events by healthcare workers. Therefore, the calculated number of hand disinfection events needed for an ideal 100% compliance is of limited accurateness and need to be considered with caution.

## Conclusions

We conclude that the tested type of dispenser provides useful trend data that can be evaluated supplementary to the data obtained form direct observations. Although not tested as such in this study, we believe that electronic devices could be useful to evaluate the long-term effect of hand hygiene promotion campaigns. Direct observations according to the ‘My five moments for hand hygiene’ approach still provide important additional information on non-compliance and quality of hand hygiene.

## Abbreviations

NICU: Neonatal intensive care unit; IQR: Interquartile range.

## Competing interests

All authors declare that they have no conflicts of interests.

## Authors’ contributions

OH, JB, JvG, and RK designed the study, WH managed and analysed the data, OH and RK wrote the manuscript and all authors contributed to data interpretation and manuscript revision and approved the final draft.

## Pre-publication history

The pre-publication history for this paper can be accessed here:

http://www.biomedcentral.com/1471-2334/12/248/prepub
